# Can Convict Cichlids (*Amatitlania siquia*) Socially Learn the Degree of Predation Risk Associated with Novel Visual Cues in Their Environment?

**DOI:** 10.1371/journal.pone.0075858

**Published:** 2013-09-24

**Authors:** Patrick M. Barks, Jean-Guy J. Godin

**Affiliations:** 1 Department of Biology, Carleton University, Ottawa, Ontario, Canada; 2 Department of Biological Sciences, University of Lethbridge, Lethbridge, Alberta, Canada; Institute of Marine Research, Norway

## Abstract

For many animals, the ability to distinguish cues indicative of predation risk from cues unrelated to predation risk is not entirely innate, but rather is learned and improved with experience. Two pathways to such learning are possible. First, an animal could initially express antipredator behaviour toward a wide range of cues and subsequently learn which of those cues are non-threatening. Alternatively, it could initially express no antipredator behaviour toward a wide range of cues and subsequently learn which of them are threatening. While the learned recognition of threatening cues may occur either through personal interaction with a cue (asocial learning) or through observation of the behaviour of social companions toward a cue (social learning), the learned recognition of non-threatening cues seems to occur exclusively through habituation, a form of asocial learning. Here, we tested whether convict cichlid fish ( *Amatitlaniasiquia* ) can socially learn to recognize visual cues in their environment as either threatening or non-threatening. We exposed juvenile convict cichlids simultaneously to a novel visual cue and one of three (visual) social cues: a social cue indicative of non-risk (the sight of conspecifics that had previously been habituated to the novel cue), a social cue indicative of predation risk (the sight of conspecifics trained to fear the novel cue), or a control treatment with no social cue. The subsequent response of focal fish, when presented with the novel cue alone, was not influenced by the social cue that they had previously witnessed. We therefore did not find evidence that convict cichlids in our study could use social learning to recognize novel visual cues as either threatening or non-threatening. We consider alternative explanations for our findings.

## Introduction

In response to predation risk, disturbance, or even novelty, many animals will exhibit some form of antipredator or avoidance behaviour such as fleeing, hiding, increased vigilance, or reduced activity [1-4]. These behavioural responses may benefit prey by reducing their immediate risk of predation, but at potential energetic and lost opportunity costs [1,3]. Assuming such costs, natural selection should favour the selective expression of antipredator behaviour during only those periods in which there is a genuine risk of predation [5,6].

For an animal to only express antipredator behaviour during periods of actual predation risk, it must first be able to assess the local risk of predation. Specifically, the individual must be able to recognize and distinguish between cues in its environment that indicate predation risk (threats) and cues unrelated to predation risk (non-threats). Such an ability may be innate [7], learned [8], or some combination of both [9].

If an animal’s ability to distinguish threats from non-threats involves learning, then two pathways to learning are possible. First, an individual could initially express weak antipredator behaviour toward a wide range of novel cues and subsequently learn which of those cues are threats (e.g. [10]). Alternatively, it could initially express strong antipredator behaviour toward a wide range of novel cues and subsequently learn which of them are not threats (e.g. [11]). When animals use learning to recognize a cue as threatening (the former pathway), learning may result either from personal interaction with the cue (asocial information, e.g. [12]), or be based on the behaviour of social companions toward that cue (social information [13]). For example, a songbird might come to fear visual cues associated with a particular raptor after narrowly escaping an attack by that raptor (asocial learning), or after hearing conspecific alarm calls in close proximity to a raptor (social learning). In contrast, examples of animals learning to recognize non-threatening cues (the latter approach) involve only asocial information (i.e. habituation [11,14,15]).

A variety of social cues can be involved in the socially learned recognition of threats. These cues include predator inspection or mobbing behaviour [16], vocal alarm calls [17,18], chemical alarm cues [19,20], and stereotypical fright responses [21]. The socially learned recognition of a threat occurs when an animal experiences one of these social cues together with a novel cue (often the novel cue is associated with a predator), and expresses increased antipredator behaviour toward that novel cue in future encounters [13]. Presumably, this type of learning is adaptive because the social cues that animals learn from are reliably indicative of predation risk. For social learning to be involved in the recognition of non-threats, there would have to exist a social cue that is reliably indicative of a lack of predation risk. One such cue might be the sight of active or actively foraging conspecifics. Reductions in activity and foraging rates are common antipredator responses in a variety of taxa [1-3]. Furthermore, in some species, the sight of active or actively foraging conspecifics can facilitate activity and foraging in nearby observers (e.g. [22]), just as the sight of conspecific fright responses can facilitate antipredator behaviour in nearby observers (e.g. [23]). The sight of active or actively foraging conspecifics may therefore be reliably indicative of a relative lack of local predation risk.

To our knowledge, no previous study has tested whether animals can learn to recognize cues in their environment as non-threatening via social information, and there have been no comparative or theoretical analyses of the evolution of asocial versus social learning with respect to predation threats (or non-threats). Therefore, we have no a priori knowledge regarding potential species or ecological characteristics that would favour an ability to socially learn about the nature of either threatening or non-threatening cues.

Here, we report on a laboratory experiment testing whether juvenile convict cichlid fish ( *Amatitlaniasiquia* ) can learn to recognize novel visual cues as either threatening or non-threatening using social information (visual cues) from conspecifics. Juvenile convict cichlids are a suitable model species to test this hypothesis given that they (i) are preyed upon by many different species in nature [24,25], (ii) respond to social cues indicative of predation risk [26], and (iii) express neophobia, an initial fear of novel stimuli [27].

## Materials and Methods

### Ethics Statement

All work reported herein was conducted in accordance with the laws of Canada and the guidelines for use of animals in research of the Canadian Council on Animal Care, and was approved by the Carleton University Animal Care Committee (protocols B10-34).

### Study Subjects

The experimental subjects were laboratory-born juvenile convict cichlids (total length = 4.0 ± 0.6 cm, mean ± SD). Our laboratory breeding stock was originally derived from, and has been periodically outbred with, wild convict cichlids collected in the Rio Cabuyo, Costa Rica. For the current study, broods were taken from their parents approximately one month after hatching and transferred to 80 l stock aquaria (2–5 broods per aquarium) containing continuously filtered, aged tap water (24–26°C), a gravel substrate, an artificial plant, and a flowerpot refuge. The aquaria were exposed to overhead fluorescent lighting on a 13 h L : 11 h D cycle. The fish were fed twice daily with frozen brine shrimp (Hikari Sales USA Inc., Hayward, CA, USA) or live brine shrimp nauplii (San Francisco Bay Brand Inc., Newark, USA) and crushed commercial food granules (Tetra Holding Inc., Blacksburg, USA), and were maintained in the same stock aquaria until the time of testing.

### Experimental Protocol

#### General Design

Each experimental trial spanned three consecutive days and was divided into three phases: training, conditioning, and recognition. In the initial training phase, a group of ‘demonstrator’ cichlids were habituated to, or trained to fear, either a ‘fat’ or a ‘skinny’ novel object (see Figure S1 for photograph). Subsequently, in the conditioning phase, a group of ‘observer’ cichlids were simultaneously exposed to one of the two novel objects and one of three social-cue treatments (habituated demonstrators, no demonstrators, or fearful demonstrators), and were thus given an opportunity to form a learned association between a novel object and a social cue. Finally, in the recognition phase, we quantified the behavioural response of the same group of observers when exposed to a novel object alone (always the skinny object) to determine whether observers learned about the novel object during the previous conditioning phase.

In this experimental design, the ‘fat object’ and ‘no demonstrator’ treatments are necessary controls that allow us to determine whether putative learning by observers results simply from prior experience with the particular novel object (e.g. sensitization), prior experience with the social cue indicative of risk or non-risk (e.g. increased or decreased baseline fear), or the prior simultaneous experience of both the novel object and social cue (true social learning). To better illustrate the inferences that can be drawn from our experimental design, we provide hypothetical results and their interpretation in Figure S2.

For both demonstrators and observers, each group (i.e. experimental unit) comprised four size-matched individuals. We chose a group of four fish as the experimental unit because (i) juvenile convict cichlids are generally inactive as singletons [28] and (ii) our pilot observations suggested that solitary individuals or groups of fewer than four individuals generally remained motionless and under shelter for the duration of the trial, which would make it difficult to assess their response to a novel object. Similar effects of group size on activity budgets and foraging rates are common in a variety of species [29]. Our use of groups as an experimental unit necessitates the assumption that learning, a property of individuals, can be understood in terms of the average behaviour expressed by a group of individuals.

For each of the six object × social cue combinations, we conducted 15 replicate trials for a total sample size of 90.

#### Apparatus

**Figure 1:**
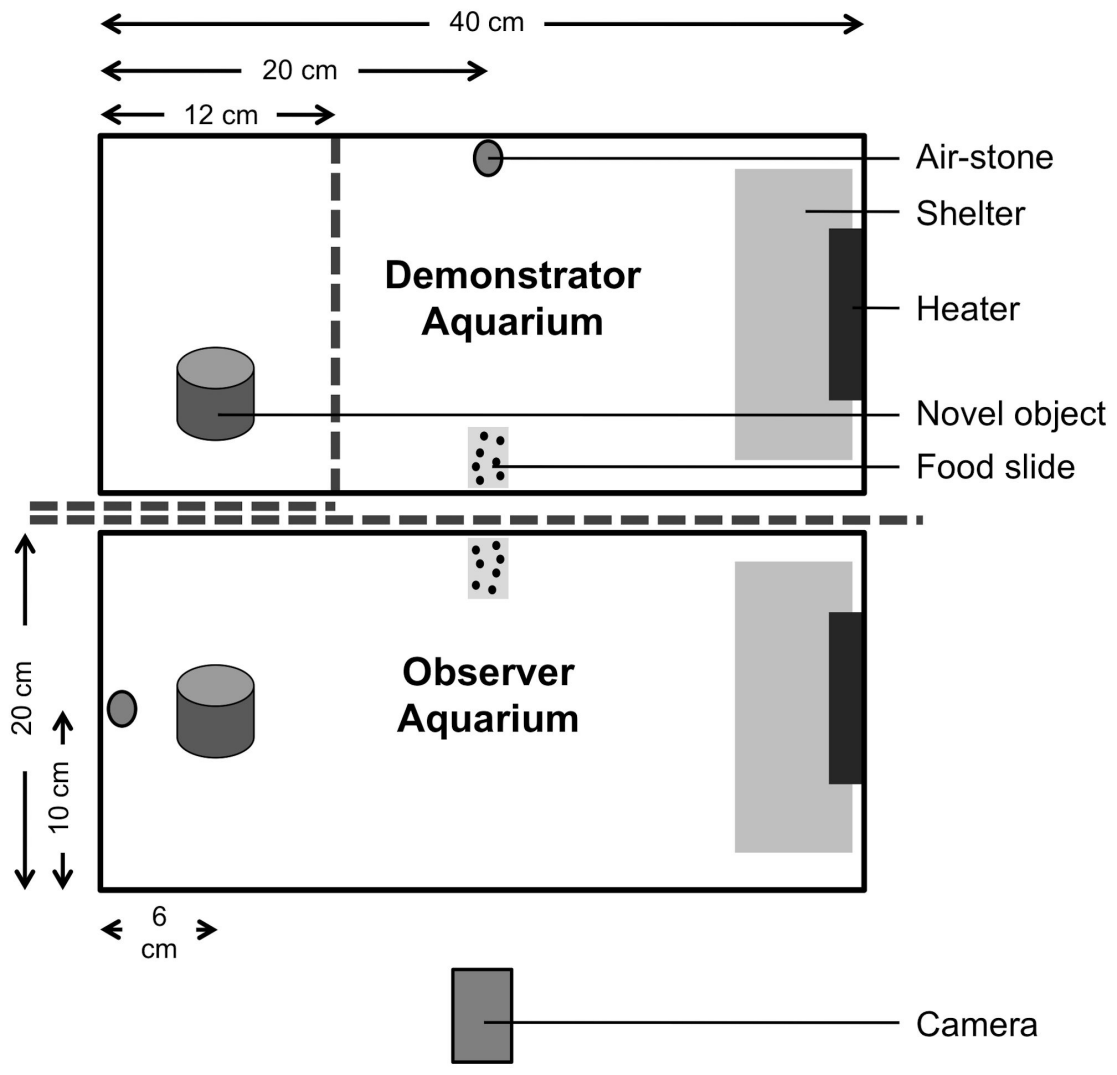
Experimental apparatus. A top-view schematic representation of the experimental apparatus used in the current study. The dashed lines represent three opaque partitions that were each remotely removed at different points during an experimental trial, as described in the text. Likewise, the novel objects (see Figure S1) and food slides were added to and removed from the apparatus as described in the text. We note that, within the observer aquarium, the novel object and food slide were only present during portions of the recognition phase, and were not present during the conditioning phase.

The experimental apparatus consisted of two adjacent 20 l aquaria, one for demonstrators and the other for observers (Figure 1). Both aquaria contained a gravel substrate, a heater, an air-stone, and a shelter as a potential refuge (an 8 × 15 cm sheet of white Plexiglas raised 5 cm above the substrate via three glass ‘legs’). The demonstrator aquarium also contained a remotely moveable opaque Plexiglas partition that divided the aquarium into a large compartment for the demonstrators and a smaller compartment where the novel object was placed (see Figure 1). Two additional opaque partitions separated the demonstrator aquarium from the observer aquarium. The longer partition completely separated the two aquaria, whereas a second shorter partition prevented observers from seeing into the smaller compartment of the demonstrator aquarium that contained the novel object. Each of the partitions could be remotely removed and replaced at different points during an experimental trial, as described below.

Each test aquarium also contained a number of items that were present for only certain portions of a trial (as described in the following subsections), including a food patch (microscope slide covered with crushed commercial food granules affixed to it via a thin coating of gelatin), and a novel object at the end of the aquarium opposite the shelter. The fat novel object was an 8 × 6 cm steel cylinder (EMT Set Screw Connector, Model BC-5016, Thomas and Betts Corporation, Memphis, TN, USA) and the skinny novel object was a 10 × 4 cm brass cylinder (Rough Brass Sink Tailpiece, Model R-812-4RB, Oakville Stamping and Bending, Oakville, ON, Canada) (Figure S1). The objects were chosen arbitrarily with the intention that they would be entirely novel to the cichlids, and be similar enough to illicit similar behavioural responses, but sufficiently different to prevent a learned generalization from one object to the other (e.g. [30,31]).

#### Acclimatization

The day before an experimental trial began, a group of observers and a group of demonstrators were transferred from stock aquaria into their respective test aquaria (assignment was randomized with respect to treatment and object) between 16:00–19:00 hrs and left undisturbed to acclimatize overnight. The following morning (Day 1 of the experiment), a single food slide was placed into each experimental aquarium between 09:00–11:00 hrs. All experimental fish were accustomed to foraging from such a food patch as similar food slides were regularly placed in the stock aquaria. Throughout the acclimatization phase, demonstrators and observers remained visually isolated from each other as an opaque partition was placed between their respective aquaria.

For a given trial, all four fish within a test aquarium (i.e. all four observers or all four demonstrators) were taken from the same stock aquarium, but demonstrators and observers within a given trial originated from different stock aquaria. Therefore, individuals were always familiar with and possibly akin to the other individuals in the same test aquarium, but were never familiar with or akin to the individuals in the adjacent aquarium. This process of ensuring demonstrators and observers came from different stock aquaria controlled for any potential effect of social familiarity on behaviour [32]. The selection and size matching of experimental subjects from stock aquaria was performed haphazardly and without actually measuring the fish prior to experimentation to minimize stress (fish were measured at the end of each experimental trial). The total body length of demonstrators and observers was 4.1 ± 0.3 cm and 3.9 ± 0.4 cm, respectively. For the 60 trials that included demonstrators, the mean (± SD) difference in mean total body length between paired groups of demonstrators and observers (mean body length of the four demonstrators – mean body length of the four observers) was 0.2 ± 0.3 cm.

#### Training Phase [Demonstrators Only]

The purpose of the training phase was to generate groups of demonstrators that would respond in one of two manners to a novel object (half the groups were trained with the fat object and the other half with the skinny object). Demonstrators in the habituated treatment were trained not to respond to the novel object, potentially indicating to observers that the object was safe, whereas demonstrators in the fearful treatment were trained to exhibit an avoidance or antipredator response when the object was presented, potentially indicating to observers that the novel object represented a threat. The third treatment was a control with no demonstrators present and therefore no training.

The training phase comprised three separate training sessions conducted between 15:00–17:00 hrs on Day 1, 08:00–10:00 hrs on Day 2, and 11:00–13:00 hrs on Day 2, respectively. Demonstrators and observers remained visually isolated from each other throughout the training phase via the same opaque partitions previously described. To train demonstrators in the habituated treatment, we remotely raised the opaque partition previously separating them from the novel object in their test aquarium, and then left the demonstrators alone until 1 h prior to the beginning of the next session, at which time the partition was gently replaced. The total time that habituated demonstrators were exposed to the novel object was approximately 20 h, 55% of which was at night.

To train demonstrators in the fearful treatment, we remotely raised the opaque partition separating demonstrators from the novel object and then repeatedly passed a small dipnet through their aquarium in a standardized manner every 30 s for 3 min, after which time the partition was replaced. Each pass of the dipnet lasted approximately 5 s and consisted of the following sequential steps: the experimenter inserted the dipnet into the demonstrator aquarium near the novel object, rapidly moved it across the length of the aquarium up to the shelter, then slowly moved the dipnet back toward the novel object, and repeated this process once more. Throughout this process, the experimenter was visually isolated (i.e. hidden) from the demonstrators. The decision to use a dipnet to train demonstrators in the fearful treatment was based on our personal observation that cichlids avoided the dipnet more strongly than any other object introduced into their aquaria, including realistic fish predator models. Others have similarly used dipnets to simulate predation risk and test for antipredator responses in fishes (e.g. [33]).

Pilot trials conducted prior to the experiment suggested that the above training regime was effective. Fearful demonstrators reduced their activity and foraging rates and moved inside the shelter upon post-training presentation of the novel object they were trained with, whereas habituated demonstrators did not respond to the object following training. We acknowledge the possibility that demonstrators were not responding to the novel object per se, but rather to the raising of the partition. However, this would not affect our experimental design or interpretation of results because (i) observers in all three treatments witnessed a novel object and the raising of a partition during the conditioning phase (the only thing that differed between treatments was demonstrator behaviour), and (ii) during the recognition phase, the novel object was presented to observers without the use of a partition.

#### Conditioning Phase [Observers and Demonstrators]

The purpose of the conditioning phase was to present observers with an opportunity to socially learn about a novel object based on the behavioural response of trained demonstrators toward that object. Five minutes prior to the start of conditioning, we removed the larger of the two opaque partitions separating the demonstrators and observers, thus allowing the two groups to see each other (in the two social cue treatments that involved demonstrators) and to acclimatize to their new surroundings. The other two partitions remained in place so that both demonstrators and observers were visually isolated from the compartment within the demonstrator aquarium that contained the novel object. Immediately after removing the partition, we placed a food slide in the centre of the demonstrator aquarium (see Figure 1). Typically, demonstrators would begin foraging within seconds and observers would rapidly swim alongside the wall of their own aquarium nearest the adjacent demonstrators and food slide.

The conditioning phase was 6 min in duration and was conducted between 15:00–17:00 hrs on Day 2 of each experimental trial. During this phase, the behaviour of both observers and demonstrators (where present) was videotaped from a side-on-view (see Figure 1) using a digital HD video camera (Sony HandyCam HDR-HC7, Sony Electronics Inc., San Diego, CA, USA). Conditioning began 5 min after the removal of the long partition (described above), and consisted of a 3-min pre-object period to establish the baseline behaviour of both observers and demonstrators, followed by the remote removal of the remaining two partitions and a 3-min post-object period, during which time observers could witness the behaviour of demonstrators toward the novel object. At the end of the post-object period, the long partition separating observers from demonstrators was gently replaced, and demonstrators had no further role in the experiment. Note that our conditioning regime restricted information transmission between observers and demonstrators to visual cues only. This setup allowed us to isolate the specific modality involved in potential learning events, and corresponded with the modality of our proposed social indicator of non-risk (the sight of active and actively-foraging conspecifics). Note also that, apart from not seeing demonstrators within the demonstrator aquarium, observers in the ‘no-demonstrator’ social cue treatment were conditioned in exactly the same manner as those in the other two social cue treatments.

#### Recognition Phase [Observers Only]

The purpose of the recognition phase was to assess the response of observers toward a novel object (always the skinny object) to determine if they socially learned about the object during the previous conditioning phase. We chose to present only one of the novel objects during the recognition phase for logistical and ethical reasons; a balanced design (i.e. half of the observer groups exposed to one object during the recognition phase, and half to the other) would have required twice as many trials and therefore twice as many fish to achieve equivalent statistical power. The recognition phase was conducted between 09:00–12:00 hrs on Day 3 of each trial, and was videotaped in the same manner as the conditioning phase. Approximately 1 min prior to the beginning of the recognition phase, a food slide was introduced into the observer aquarium halfway between the shelter and the location of the novel object (see Figure 1). The recognition phase began after observers initiated foraging and consisted of a 3-min pre-object period, followed by a 3-min post-object period (total trial time = 6 min). At 3 min into the trial, the skinny object was remotely and slowly lowered into the aquarium via a pulley system utilizing monofilament nylon line. The object had until that point been hidden behind the overhead lighting, approximately 1 m above the experimental aquarium. Following the recognition phase, all fish were digitally photographed (for length measurement) and then transferred to new stock aquaria or euthanized with an overdose of the anesthetic MS-222. None of the experimental fish (including both demonstrators and observers) were used in more than one trial.

### Behavioural Measures

To understand whether observers socially learned to recognize a novel object as either threatening or non-threatening, we modeled their behavioural response toward the skinny object during the recognition phase as a function of the treatment and object they experienced during the conditioning phase. The two behavioural measures that we focused on were change in shelter use and change in foraging rate between the pre- and post-object periods of the recognition phase. Increases in shelter use and decreases in foraging rate are both commonly used measures of antipredator behaviour in convict cichlids [26,34,35] and in animals in general [1-3].

Our two behavioural measures were quantified based on an analysis of image frames extracted from video footage (using the screen capture function in Mac OS X v10.5.8; Apple Inc., Cupertino, CA, USA) at 5-s intervals for the duration of the recognition phase of each trial. Given that the pre- and post-object periods of the recognition phase were each 3 min in duration, we extracted a total of 72 image frames for each trial: frames 1–36 represented the pre-object period, and frames 37–72 represented the post-object period. For each image frame of each trial, we recorded the number of fish that were observed under the shelter and the number of fish that appeared to be foraging (since there were four fish in each experimental unit, these measures range from 0-4). An individual was scored as being ‘under shelter’ if its two-dimensional centre of mass (estimated visually from the extracted image frames) was within the vertical and horizontal limits of the artificial shelter, whereas ‘foraging’ was defined as being within one body length of and oriented toward the food slide. Pre-object and post-object shelter use for a given trial was defined as the mean number of fish observed under shelter per video frame for the pre-object or post-object period, respectively. Similarly, the pre-object and post-object foraging rate was calculated as the mean number of fish observed foraging per video frame for the pre-object or post-object period, respectively. The person (PMB) quantifying these behavioural measures was blind to the treatment and object that observers experienced during the conditioning phase.

The response of observer fish to the novel object in each of the three social cue treatments was calculated as follows. For each trial, we quantified both the change in shelter use by observer fish as post-object shelter use minus pre-object shelter use, and the change in their foraging rate as post-object foraging rate minus pre-object foraging rate. A positive change (increase) in shelter use and negative change (decrease) in foraging rate were taken to be indicative of fear or avoidance of the novel object.

To ensure that demonstrators behaved as anticipated during conditioning, we quantified change in shelter use and foraging rate by demonstrators during the conditioning phase in exactly the same manner as for observers during the recognition phase. This was done only for the habituated and fearful treatments, as there were no demonstrators in the control treatment.

Finally, to test whether observers responded to the behaviour of demonstrators in real time during the conditioning phase, we quantified change in shelter use by observers during the conditioning phase as described above. We were not able to quantify changes in the foraging rate of observers during conditioning because there was no food slide in the observer aquarium during the conditioning phase.

### Statistical Analyses

All analyses were performed using R 2.12.0 [36]. To determine whether the behaviour of demonstrators during conditioning truly reflected their training, we modeled their behavioural responses (changes in shelter use and foraging rate) toward the novel object during the conditioning phase as a function of the object they were presented with and their treatment. We used analysis of variance (ANOVA) to test our expectation that demonstrators in the fearful treatment should exhibit a greater increase in shelter use and decrease in foraging rate than those in the habituated treatment.

To test whether observers responded to the social cue that they witnessed during the conditioning phase, we modeled using ANOVA change in shelter use by observers during the conditioning phase as a function of the novel object and social cue that they were conditioned with. Additionally, to more directly test for associations between the behaviour of observers and the demonstrators they witnessed during conditioning, we modeled using analysis of covariance (ANCOVA) change in shelter use by observers during the conditioning phase as a function of the novel object they were presented with and the behavioural response (change in shelter use) of the demonstrators they witnessed. This latter analysis excluded observers in the control treatment as they did not witness demonstrators.

To ascertain whether observers socially learned to recognize a novel object as either threatening or non-threatening, we modeled their behavioural responses (changes in shelter use and foraging rate) toward the skinny object during the recognition phase as a function of the object and treatment they experienced during the conditioning phase, while controlling for differences in the body length of observers using ANCOVA. We included body length as a covariate in these analyses because many behavioural and physiological traits vary with body size in fishes (e.g. [37-39]). If observers socially learned about a novel object based on the behaviour of demonstrators during the conditioning phase, we would expect a significant interaction between object and treatment in the aforementioned analysis. Specifically, observers conditioned with the skinny object and fearful demonstrators should subsequently react more strongly toward the skinny object than other groups, whereas observers conditioned with the skinny object and habituated demonstrators should subsequently react less strongly toward the skinny object than other groups.

Finally, to determine whether the behaviour of demonstrators during the conditioning phase influenced the subsequent behaviour of observers during the recognition phase (irrespective of the specific treatment), we modeled the change in shelter use (or foraging rate) of observers during the recognition phase as a function of the particular novel object they experienced during conditioning and the change in shelter use (or foraging rate) of the demonstrators they witnessed during the conditioning phase, whilst controlling for the body length of observers using ANCOVA. Because there were no demonstrators in the control treatment, the latter analyses were limited to observers in the fearful and habituated treatments only. If observers learned about an object based on the behaviour of demonstrators during the conditioning phase, we would expect a positive correlation between the behaviour of demonstrators during the conditioning phase and the subsequent behaviour of observers during the recognition phase, but only among those observers that were conditioned with the skinny object (i.e. the only object that was presented during the recognition phase).

To ensure that the results we obtained were not simply a function of the specific time-interval chosen for the pre- and post-object periods of each phase (i.e. 3 min), we repeated all of the analyses described above using behavioural measures based on 1-min pre- and post-object periods instead of 3-min periods. Results based on 1-min periods were in all cases similar to (and strongly correlated with) those based on 3-min periods; consequently, we do not specifically discuss results of the 1-min analyses below. Instead, figures based on 1-min pre- and post-object periods are included as Supporting Information (Figures S3, S4, S5 and S6).

For all statistical analyses reported herein, we visually inspected Q-Q and residual plots to ensure that the assumptions of parametric statistical tests were met.

## Results

### Conditioning Phase

**Figure 2:**
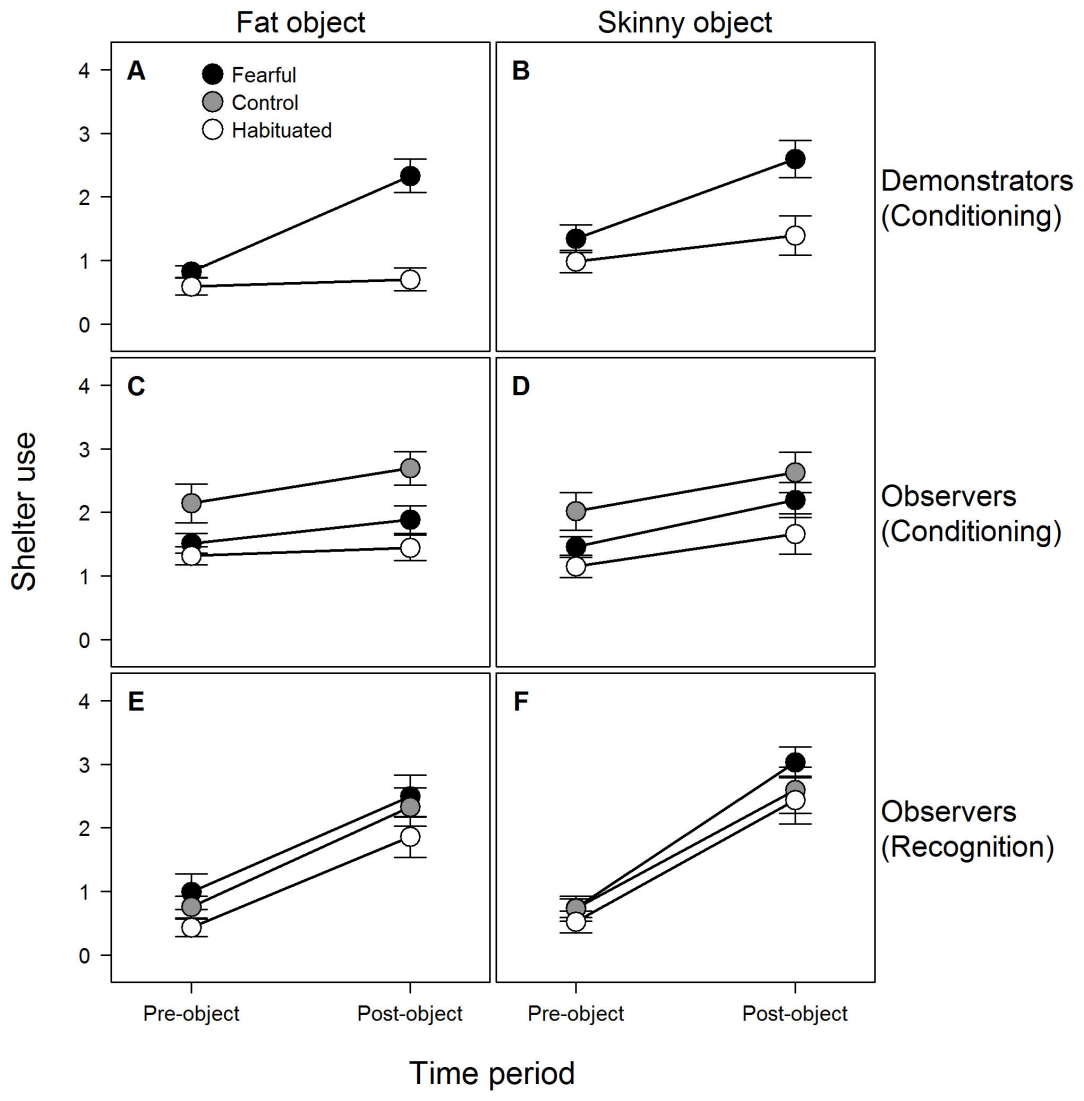
Changes in shelter use in response to a novel object. Mean (± SE) shelter use by demonstrators during the conditioning phase (A, B) and the corresponding observers during either the conditioning phase (C, D) or recognition phase (E, F). During conditioning, demonstrators that had previously been either habituated to (open circles) or made fearful of (black circles) either the fat (A) or skinny (B) object were presented with the same object that they had been trained with while observers (C, D) witnessed their behaviour from adjacent aquaria. In the recognition phase, observers that, during conditioning, had witnessed either fearful demonstrators (black circles), no demonstrators (grey circles), or habituated demonstrators (open circles) reacting to either the fat (E) or skinny (F) object, were all presented with the skinny object. In each panel, pre-object scores reflect behaviour during the three minutes prior to presentation of an object, whereas post-object scores reflect behaviour during the three minutes following object presentation.

**Figure 3:**
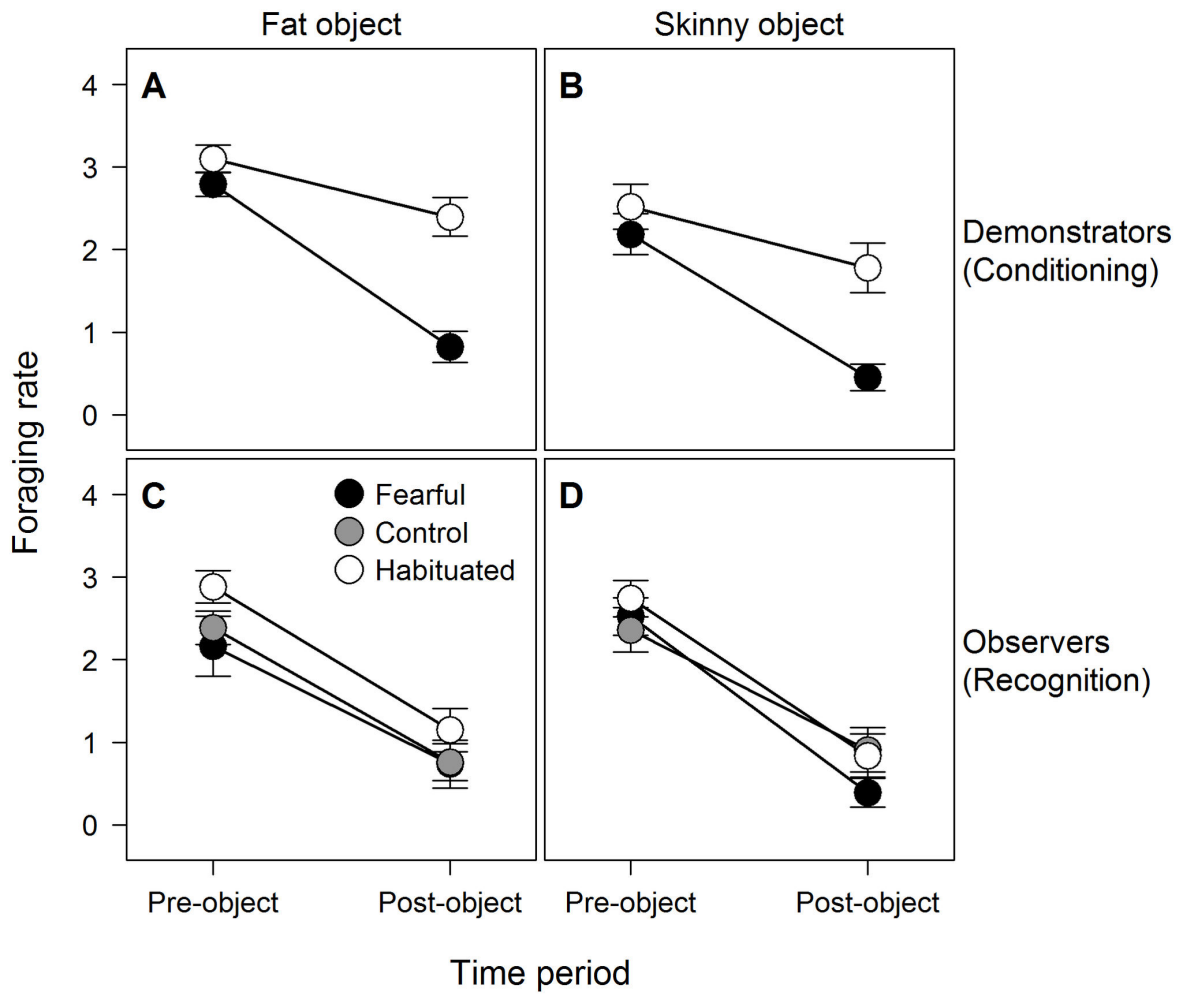
Changes in foraging rate in response to a novel object. Mean (± SE) foraging rate of demonstrators during the conditioning phase (A, B) and the corresponding observers during the recognition phase (C, D). During conditioning, demonstrators that had previously been either habituated to (open circles) or made fearful of (black circles) either the fat (A) or skinny (B) object were presented with the same object that they had been trained with while observers witnessed their behaviour from adjacent aquaria. In the recognition phase, observers that, during conditioning, had witnessed either fearful demonstrators (black circles), no demonstrators (grey circles), or habituated demonstrators (open circles) reacting to either the fat (C) or skinny (D) object, were all presented with the skinny object. In each panel, pre-object scores reflect behaviour during the three minutes prior to presentation of an object while post-object scores reflect behaviour during the three minutes following object presentation.

During the conditioning phase, demonstrators in the fearful treatment exhibited a significantly greater increase in shelter use (*F*_1, 56_ = 30.96, *p* < 0.001; Figure 2A,B) and decrease in foraging rate (*F*_1, 56_ = 37.14, *p* < 0.001; Figure 3A,B) than demonstrators in the habituated treatment. There was no significant effect of object type on either change in shelter use (*F*_1, 56_ = 0.01, *p* = 0.91) or foraging rate (*F*_1, 56_ = 0.28, *p* = 0.60), indicating that demonstrators trained and presented with the fat object responded similarly to those trained and presented with the skinny object. These results suggest that, irrespective of the particular object, fearful demonstrators feared or avoided the novel object they were trained with more strongly than habituated demonstrators, as expected.

**Figure 4:**
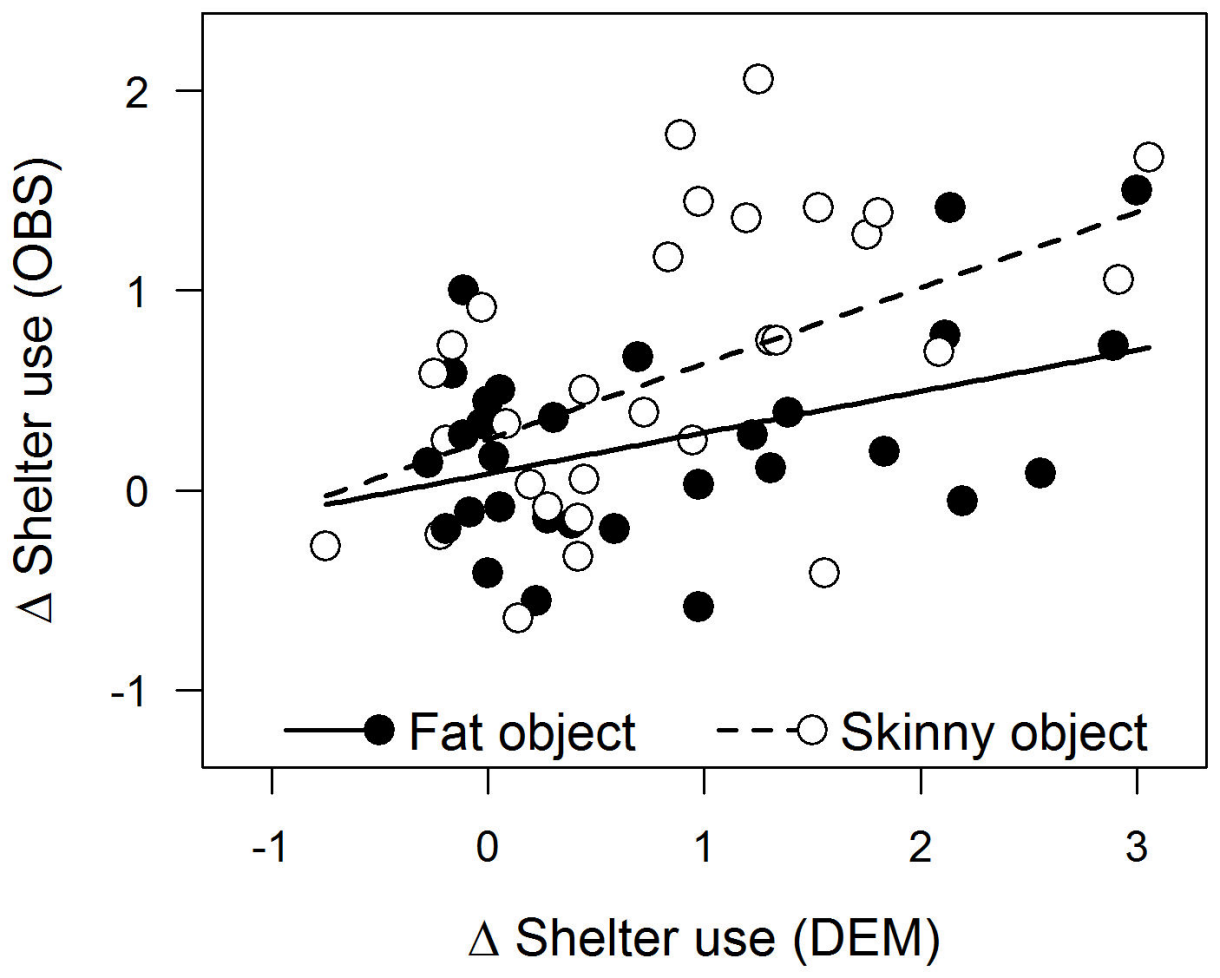
Relationship between the behaviour of demonstrators and observers during the conditioning phase. Relationship between the change (Δ) in shelter use of observers (OBS) and their corresponding demonstrators (DEM) during the conditioning phase. The depicted relationships are based on all trials for which there were demonstrators (i.e. habituated and fearful treatments combined, n = 60). Scores for groups exposed to the fat object during the conditioning phase are shown as black circles (and solid regression lines), whereas scores for those exposed to the skinny object during the conditioning phase are depicted as open circles (and dashed regression lines).

Change in shelter use by observers during the conditioning phase was significantly influenced by the object that they witnessed (*F*_1, 84_ = 4.18, *p* = 0.04), but not by the social cue (*F*_2, 84_ = 1.61, *p* = 0.21) they witnessed or the interaction between object and social cue (*F*_2, 84_ = 0.61, *p* = 0.55) (Figure 2C,D). The significant effect of object type was due to a slightly greater increase in shelter use among observers that witnessed the skinny object compared to those that witnessed the fat object (Table S1). When we excluded observers in the control treatment and tested for a direct relationship between the behaviour of observers and demonstrators during the conditioning phase, we found that change in shelter use by observers during conditioning was (i) again significantly greater among those that witnessed the skinny object compared to the fat object (*F*_1, 56_ = 7.14, *p* = 0.01), (ii) positively related to change in shelter use by demonstrators (*F*_1, 56_ = 18.04, *p* < 0.01), and (iii) not significantly influenced by the interaction between object and change in shelter use by demonstrators (*F*_1, 56_ = 2.56, *p* < 0.12) (Figure 4).

### Recognition Phase

**Figure 5:**
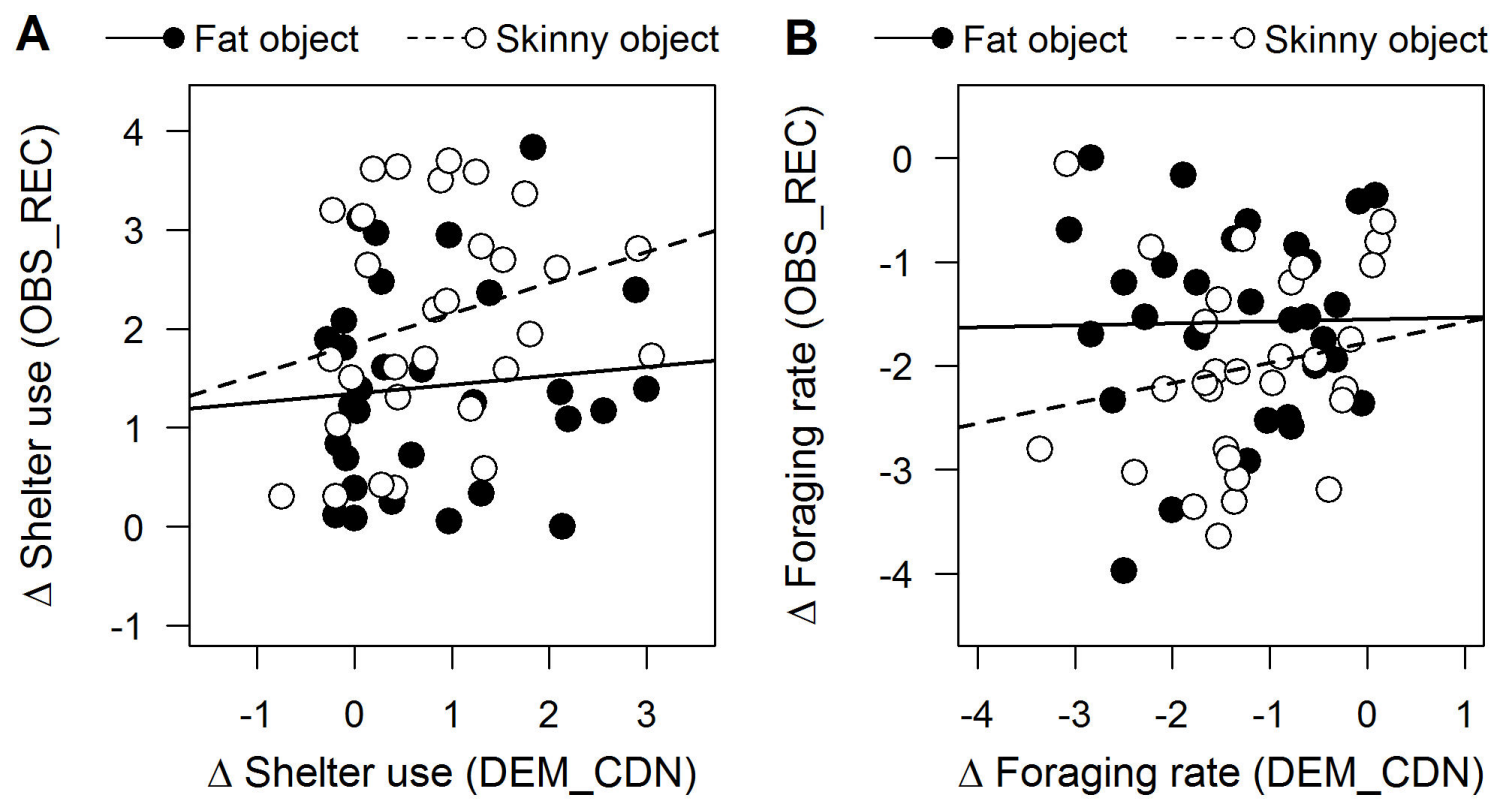
Relationship between the behaviour of demonstrators and observers during the conditioning and recognition phases, respectively. Relationship between the change (Δ) in shelter use (A) and foraging rate (B) of observers during the recognition phase (OBS_REC) and that of the corresponding demonstrators during the conditioning phase (DEM_CDN). The depicted relationships are based on all trials for which there were demonstrators (i.e. habituated and fearful treatments combined, n = 60). Scores for groups exposed to the fat object during the conditioning phase are shown as black circles (and solid regression lines), whereas scores for those exposed to the skinny object during the conditioning phase are depicted as open circles (and dashed regression lines).

During the recognition phase, change in shelter use by observers upon presentation of the skinny object was significantly influenced by the type of object presented during conditioning (*F*_1, 83_ = 5.55, *p* = 0.02), but was not significantly related to the conditioning treatment (i.e. social cue) (*F*_2, 83_ = 0.53, *p* = 0.59) nor the interaction between object and treatment (*F*_2, 83_ = 0.37, *p* = 0.69) (Figure 2E,F). The significant effect of object type was due to a slightly greater increase in shelter use among observers conditioned with the skinny object compared to those conditioned with the fat object (Table S1). When we limited our analysis to observers within the fearful and habituated treatments (i.e. only those observers that witnessed demonstrators during conditioning), change in shelter use by observers during the recognition phase was significantly influenced by both the object presented during conditioning (*F*_1, 55_ = 6.26, *p* = 0.02) and the change in shelter use by demonstrators during conditioning (*F*_1, 55_ = 4.98, *p* = 0.03), but not by the interaction between object and change in shelter use by demonstrators during conditioning (*F*_1, 55_ = 0.18, *p* = 0.67) (Figure 5A). The non-significant interaction terms described above (object × conditioning treatment, object × change in shelter use by demonstrators) are inconsistent with the occurrence of social learning among observers.

Changes in the foraging rate of observers during the recognition phase were not significantly influenced by either the object (*F*_1, 83_ = 1.30, *p* = 0.26) or treatment (*F*_2, 83_ = 0.28, *p* = 0.76) that observers experienced during the conditioning phase, nor by the interaction between object and treatment (*F*_2, 83_ = 1.65, *p* = 0.20) (Figure 3C,D). When we limited our analysis to observers within the fearful and habituated treatments (i.e. only those observers that witnessed demonstrators during conditioning), changes in the foraging rate of observers during the recognition phase were not significantly affected by object type (*F*_1, 55_ = 3.13, *p* = 0.08), changes in the foraging rate of demonstrators during conditioning (*F*_1, 55_ = 0.53, *p* = 0.47), nor by the interaction between object type and changes in the foraging rate of demonstrators during conditioning (*F*_1, 55_ = 0.27, *p* = 0.60) (Figure 5B). Similar to the above results for shelter use, the observed changes in the foraging rate of observers during the recognition phase are also inconsistent with social learning.

The variable that influenced changes in both shelter use and foraging rate during the recognition phase most strongly was the mean body length of observers, which was significantly related to observer behaviour in all four ANCOVAs pertaining to the recognition phase (Table S1). The estimated coefficients (from analyses of the full data set [i.e. all three treatments]) for the effect of mean body length on changes in shelter use and foraging rate were -1.14 (± 0.37, SE) and 0.89 (± 0.33), respectively. On average then, an increase in mean body length of observers resulted in a smaller increase in shelter use and smaller decrease in foraging rate (i.e. a reduced fear response) upon presentation of the skinny object during the recognition phase.

## Discussion

In the current study, juvenile convict cichlids did not learn to recognize novel objects as either threatening or non-threatening based on (visual) social cues. Specifically, cichlids that witnessed demonstrators behaving fearfully toward a novel object did not subsequently express increased antipredator behaviour toward that object. Likewise, cichlids that witnessed demonstrators behaving non-fearfully toward a novel object did not subsequently respond less strongly toward that object than cichlids in the relevant control group. Of course, these negative results do not necessarily imply that convict cichlids are incapable of socially learning to recognize novel cues as either threatening or non-threatening, or that all species are incapable of socially learning to recognize novel cues as non-threatening. Nonetheless, although there is considerable evidence suggesting that many animals can learn to recognize threats by attending to the behaviour of others [13], to our knowledge, there remains no evidence of any animal learning to recognize a cue in their environment as non-threatening based on the behaviour of social companions toward that cue.

It is difficult to make generalizations from negative results, especially in experimental studies of behaviour. Do our results reflect a limitation of our experimental design or a biological reality? Even assuming that the results reflect a biological reality, we are still unable to distinguish between various realities. For example, given our results, it is possible that convict cichlids are either (i) incapable of learning socially about predation threats and non-threats, (ii) capable of learning socially about predation threats and non-threats, but not the specific novel objects employed in the current study, or (iii) capable of learning socially about predation threats and non-threats, but not via the specific social cues employed in the current study. We consider each of these alternative explanations for our negative findings below.

First, assuming that convict cichlids are capable of socially learning to recognize the objects used in this study as threatening or non-threatening based on the social cues used, why might we have failed to detect such learning? One possibility is that the social cues indicative of predation risk (fearful demonstrators) and non-risk (habituated demonstrators) employed in the current study were not intense enough to facilitate learning. For example, in learning to recognize novel cues as threatening, some animals learn in proportion to the intensity of the social cue indicative of risk (threat-sensitive learning), or will only learn if the intensity of the social cue exceeds some threshold level (e.g. [10,28,40,41]). It might therefore be the case that, in the conditioning phase of our study, habituated demonstrators were still too fearful to act as a social indicator of non-risk to nearby observers, and fearful demonstrators were not fearful enough. This is a somewhat plausible explanation for our failure to detect a learned recognition of non-threats given that habituated demonstrators, despite being pre-exposed to the novel objects for a period of approximately 20 h, still exhibited small increases in shelter use and decreases in foraging rate upon presentation of the object during conditioning. However, fearful demonstrators often exhibited maximal antipredator behaviour upon presentation of the novel object during conditioning; many groups of demonstrators in the fearful treatment immediately stopped foraging and remained motionless under the shelter for the entire post-object portion of the conditioning phase. It therefore seems unlikely that our social indicator of predation risk was not intense enough to facilitate learning. A second potential explanation for our failure to detect social learning is that the period during which observers were exposed to the social cue (i.e. the 3 min post-object portion of the conditioning phase) may have been too short. This explanation is somewhat unlikely given that, in the context of a predation event or social encounter, three minutes is a relatively long time. More importantly, the socially learned recognition of threatening cues often occurs within a matter of minutes [42,43]. A final potential explanation for our negative results – still assuming that convict cichlids are in fact capable of learning about (via) the novel (social) cues used in this study – is that observers were never familiar with or akin to the demonstrators that they witnessed during conditioning. In some species, individuals recognize and preferentially associate with conspecifics on the basis of familiarity or kinship [44]. It seems plausible that individuals might also preferentially devote attention to and/or learn from conspecifics they are familiar with or akin to.

Second, it is possible that convict cichlids are capable of learning about predation threats and non-threats via social information, but are not capable of learning about the specific objects used in the current study. Intuitively, an animal could benefit from learning to fear any cue genuinely associated with predation risk that it does not innately fear, including visual, auditory, and olfactory stimuli associated with any potential predator. Likewise, an animal could benefit from learning not to fear any cue that is not genuinely associated with predation risk that it does innately fear. In practice, however, many animals are also capable of learning to fear a wide range of cues that are not genuinely associated with predation risk, such as plastic disks [45], synthetic odours [21], flashing lights [46], artificial sounds [47], and the sight of non-predator species [48]. Similarly, most animals can learn not to fear a wide range of stimuli, including cues that *are* genuinely associated with predation risk (e.g. [11]). Therefore, it is unlikely that convict cichlids have the ability to socially learn about threatening and/or non-threatening cues, but not the cues used in the current study (our skinny object was long and cylindrical, roughly similar to the elongated shape of a fish).

A third possible explanation for our negative results is that convict cichlids are capable of socially learning to recognize cues as either threatening or non-threatening, but are not capable of learning via the specific social cues employed in the current study. Are there different social cues that more reliably indicate predation risk or non-risk? We suggest that chemical alarm cues are another potential candidate for a social cue that reliably indicates predation risk. Chemical alarm cues are released by a variety of aquatic animals (including convict cichlids) upon physical damage to the epidermis, usually following a predation event [48]. As such, these cues are potentially a completely reliable indicator of a nearby predation event, and are therefore a good candidate for a social cue involved in the learned recognition of predation threats [48]. However, unlike the social cue employed in the current study (the sight of fearful conspecifics), chemical alarm cues only indicate that a predation event has already occurred and are potentially only transmitted to animals downstream from the predation event. We contend that chemical alarm cues and the sight of fearful conspecifics are both likely to be reliable indicators of predation risk. In the current study, we focused only on the visual social cue because it corresponded with our proposed social indicator of non-risk (the sight of active and actively-foraging conspecifics), which we argue is the only candidate for a reliable social indicator of non-risk. It is important here to emphasize that, for a cue to be a reliable indicator of non-risk, it should (i) never or only rarely be present during periods of relatively high predation risk, and (ii) frequently be present during periods of relatively low predation risk. The *absence* of chemical alarm cues is probably not a reliable indicator of risk because, even though this indicator will frequently be ‘present’ during periods of low predation risk, it may sometimes be present during periods of high predation risk (e.g. a predator is nearby but has not yet attacked, or has attacked a downstream conspecific). We therefore suggest that, given the abundant evidence for a negative relationship between activity/foraging and predation risk in animals [1-3], the sight of active or actively foraging conspecifics is the most reliable social indicator of non-risk.

If we now assume that our negative results were not related to limitations of our experimental design or our choice of novel objects or social cues, then we can consider why convict cichlids would be incapable of socially learning to recognize threats and non-threats. For the sake of brevity, we limit this discussion to the socially learned recognition of non-threatening cues since this was the novel aspect of our study. We suggest that three characteristics are required for such an ability to evolve within a given lineage. First, the lineage must exhibit some degree of neophobia; otherwise a learned decrease in antipredator behaviour would not be possible. Second, the expression of antipredator behaviour must have inherent costs so that there is a benefit to learning about non-threats. Third, members of the lineage must have exposure to social cues that are reliably indicative of non-risk. The first two criteria are satisfied in many animal species (neophobia [11,14,27,49]; costly antipredator behaviour [1-3]). Whether the third criterion is sufficiently satisfied in any species remains unknown. It has previously been proposed that social learning is an adaptation for social living [50], which leads to the commonly held assumption that, among species, the propensity for social learning positively correlates with sociality. However, there is little empirical evidence for such a relationship [51], and social learning has been documented in at least one non-social species [52]. We therefore have no reason to believe that a high degree of sociality will itself favour the socially learned recognition of non-threatening cues.

Given that our first two criteria (neophobia and costly antipredator behaviour) for the evolution of socially learned recognition of non-threats are satisfied in many species, it would seem that the best biological explanation for the lack of evidence of socially learned recognition of non-threatening cues is that the third criterion – exposure to social cues that are reliably indicative of non-risk – is rarely satisfied. The observation that many animals, including the convict cichlid, can learn to recognize non-threats via asocial information (i.e. habituation [11,14,53]), but not social information, suggests that social indicators of non-risk must either be less abundant or less reliable than asocial indicators. In the context of learning about non-threats, it may be that social cues are by their nature less reliable than asocial cues [54,55,56]. For example, the social indicator of non-risk used in the current study was the sight of active and actively foraging conspecifics. Although animals generally decrease their activity and foraging rates under increased risk of predation [1-3], the reliability of this social cue may depend on the experience, physiological state, and risk-assessment ability of the individual from whom the cue derives. For example, a nearby social companion may only respond to a threatening cue if that individual detects the cue, recognizes the cue as a threat, and is motivated to respond, which will not always be the case. It is therefore possible that high activity and foraging rates, or any other cues deriving from the behaviour of social companions, are not sufficiently indicative of low local predation risk.

Although we did not find evidence for socially learned recognition of non-threatening (nor threatening) cues in juvenile convict cichlids, we suggest, following Ferrari and Chivers [57], that the ability to learn about non-threats is an underappreciated aspect of predation risk assessment in wild animals. Some animals even appear to learn about predation risk exclusively via decreases in antipredator behaviour toward cues that are learned not to be threatening rather than increases in antipredator behaviour toward cues that are learned to be threatening (e.g. [11,14,53]). For example, Deecke et al. [53] reported that harbour seals ( *Phoca* *vitulina* ) initially express antipredator behaviour toward all underwater calls from killer whales, but selectively habituate to the calls of fish-eating killer whale populations. The taxonomically widespread occurrence of neophobia and habituation (e.g. [4,11,14,15,27,49]) suggests that, like harbour seals, many animals will initially express antipredator behaviour (to varying degrees) toward a wide range of novel cues encountered in their environment and allow individual experience to slowly dictate which cues are safe. We suggest that a thorough understanding of predation risk assessment requires an understanding of how animals learn about both threatening and non-threatening cues, and how these two forms of learning might interact.

## Supporting Information

Table S1 **Parameter estimates from fitted ANOVA or ANCOVA models.**
(DOC)

Figure S1 **Digital photograph of the two novel objects presented to experimental fish in the current study.** The so-called ‘fat object’ was a 8 × 6 cm (height × width) steel cylinder, and the ‘skinny object’ was a 10 × 4 cm brass cylinder.
(TIFF)

Figure S2 **Schematic representation of some of the possible outcomes of the recognition phase.** To test for various forms of learning, we considered the main and interactive effects of object and social cue experienced during the conditioning phase on change in shelter use by observers during the recognition phase. Regardless of the object and social cue they witnessed during the conditioning phase, we expected all observers to exhibit some increase in shelter use (and decrease in foraging rate) between the pre-object and post-object periods of the recognition phase, because presentation of the skinny object necessarily entailed some degree of disturbance. (A) Hypothetical evidence of cichlids socially learning to fear a novel object. Cichlids conditioned with the skinny novel object and demonstrators fearful of that object subsequently exhibit greater fear (increased shelter use) when presented with the skinny object than relevant controls. (B) Hypothetical evidence of some cichlids socially learning to fear a novel object and others socially learning not to fear the novel object. The smaller increase (compared to relevant controls) in shelter use by cichlids conditioned with the skinny object and demonstrators habituated to that object is consistent with those cichlids socially learning not to fear the skinny object. (C) A hypothetical effect of the type of social cue presented during the conditioning phase that occurs regardless of the object presented during the conditioning phase. This result would not be indicative of cichlids socially learning about a particular novel object, but rather would suggest that cichlids witnessing fearful demonstrators during conditioning subsequently react more fearfully to a range of stimuli.
(TIFF)

Figure S3 **Changes in shelter use in response to a novel object, based on 1-minute pre- and post-object periods.** Mean (± SE) shelter use by demonstrators during the conditioning phase (A, B) and the corresponding observers during either the conditioning phase (C, D) or recognition phase (E, F). During conditioning, demonstrators that had previously been either habituated to (open circles) or made fearful of (black circles) either the fat (A) or skinny (B) object were presented with the same object that they had been trained with while observers (C, D) witnessed their behaviour from adjacent aquaria. In the recognition phase, observers that, during conditioning, had witnessed either fearful demonstrators (black circles), no demonstrators (grey circles), or habituated demonstrators (open circles) reacting to either the fat (E) or skinny (F) object, were all presented with the skinny object. In each panel, pre-object scores reflect behaviour during the 1-min period prior to presentation of an object, whereas post-object scores reflect behaviour during the 1-min period following object presentation.
(TIFF)

Figure S4 **Changes in foraging rate in response to a novel object, based on 1-minute pre- and post-object periods.** Mean (± SE) foraging rate of demonstrators during the conditioning phase (A, B) and the corresponding observers during the recognition phase (C, D). During conditioning, demonstrators that had previously been either habituated to (open circles) or made fearful of (black circles) either the fat (A) or skinny (B) object were presented with the same object that they had been trained with while observers witnessed their behaviour from adjacent aquaria. In the recognition phase, observers that, during conditioning, had witnessed either fearful demonstrators (black circles), no demonstrators (grey circles), or habituated demonstrators (open circles) reacting to either the fat (C) or skinny (D) object, were all presented with the skinny object. In each panel, pre-object scores reflect behaviour during the 1-min period prior to presentation of an object while post-object scores reflect behaviour during the 1-min period following object presentation.
(TIFF)

Figure S5 **Relationship between the behaviour of demonstrator and observers during the conditioning phase, based on 1-minute pre- and post-object periods.** Relationship between the change (Δ) in shelter use of observers (OBS) and their corresponding demonstrators (DEM) during the conditioning phase. The depicted relationships are based on all trials for which there were demonstrators (i.e. habituated and fearful treatments combined, n = 60). Scores for groups exposed to the fat object during the conditioning phase are shown as black circles (and solid regression lines), whereas scores for those exposed to the skinny object during the conditioning phase are depicted as open circles (and dashed regression lines).
(TIFF)

Figure S6 **Relationship between the behaviour of demonstrators and observers during the conditioning and recognition phases, respectively, based on 1-minute pre- and post-object periods.** Relationship between the change (Δ) in shelter use (A) and foraging rate (B) of observers during the recognition phase (OBS_REC) and that of the corresponding demonstrators during the conditioning phase (DEM_CDN). The depicted relationships are based on all trials for which there were demonstrators (i.e. habituated and fearful treatments combined, n = 60). Scores for groups exposed to the fat object during the conditioning phase are shown as black circles (and solid regression lines), whereas scores for those exposed to the skinny object during the conditioning phase are depicted as open circles (and dashed regression lines).
(TIFF)
