# Supplementary material for: Can Convict Cichlids (Amatitlania siquia) Socially Learn the Degree of Predation Risk Associated with Novel Visual Cues in Their Environment?
Source: PLoS One. 2013 Sep 24;8(9):e75858. doi: 10.1371/journal.pone.0075858 (PMC3782494; doi:10.1371/journal.pone.0075858)
Supplement: Table S1 — Parameter estimates from fitted ANOVA or ANCOVA models. (DOC) [file pone.0075858.s001.doc]

Table S1. Parameter estimates from fitted AN(C)OVA models.

|  |  | **Response Variable** | | | |
| --- | --- | --- | --- | --- | --- |
|  |  | **∆ Shelter use** | | **∆ Foraging rate** | |
| **Focal Group, Phase** | **Parameter** | **Estimate** | **SE** | **Estimate** | **SE** |
| Demonstrators, Conditioning | Object^a^: Skinny | 0.30 | 0.29 | -0.04 | 0.26 |
|  | Treatment^b^: Fearful | **1.40** | **0.29** | **-1.26** | **0.26** |
|  | Object: Skinny × Treatment: Fearful | -0.55 | 0.40 | 0.27 | 0.37 |
| Observers, Conditioning | Object^a^: Skinny | **0.06** | **0.23** | n/a | n/a |
|  | Treatment^c^: Habituated | -0.42 | 0.23 | n/a | n/a |
|  | Treatment^c^: Fearful | -0.18 | 0.23 | n/a | n/a |
|  | Object: Skinny × Treatment: Habituated | 0.32 | 0.33 | n/a | n/a |
|  | Object: Skinny × Treatment: Fearful | 0.30 | 0.33 | n/a | n/a |
| Observers (all treatments), Recognition | Mean body length of observers | **-1.14** | **0.37** | **0.89** | **0.33** |
|  | Object^a^: Skinny | **0.32** | **0.37** | 0.16 | 0.34 |
|  | Treatment^c^: Habituated | -0.26 | 0.38 | -0.02 | 0.34 |
|  | Treatment^c^: Fearful | -0.15 | 0.37 | 0.27 | 0.34 |
|  | Object: Skinny × Treatment: Habituated | 0.13 | 0.53 | -0.29 | 0.48 |
|  | Object: Skinny × Treatment: Fearful | 0.44 | 0.53 | -0.85 | 0.48 |
| Observers (habituated and fearful treatments only), Recognition | Mean body length of observers | **-1.44** | **0.45** | **0.88** | **0.41** |
|  | Object^a^: Skinny | **0.54** | **0.34** | -0.24 | 0.42 |
|  | ∆ Behaviour by demonstrators during conditioning | **0.24** | **0.19** | 0.04 | 0.18 |
|  | Object: Skinny × ∆ Behaviour by demonstrators during conditioning | 0.11 | 0.27 | 0.14 | 0.27 |

Terms in boldface were significant at α = 0.05

^a^ Reference category = fat object

^b^ Reference category = habituated treatment

^c^ Reference category = control treatment
