# Supplementary figures and images for: Can Convict Cichlids (Amatitlania siquia) Socially Learn the Degree of Predation Risk Associated with Novel Visual Cues in Their Environment?
Source: PLoS One. 2013 Sep 24;8(9):e75858. doi: 10.1371/journal.pone.0075858 (PMC3782494; doi:10.1371/journal.pone.0075858)

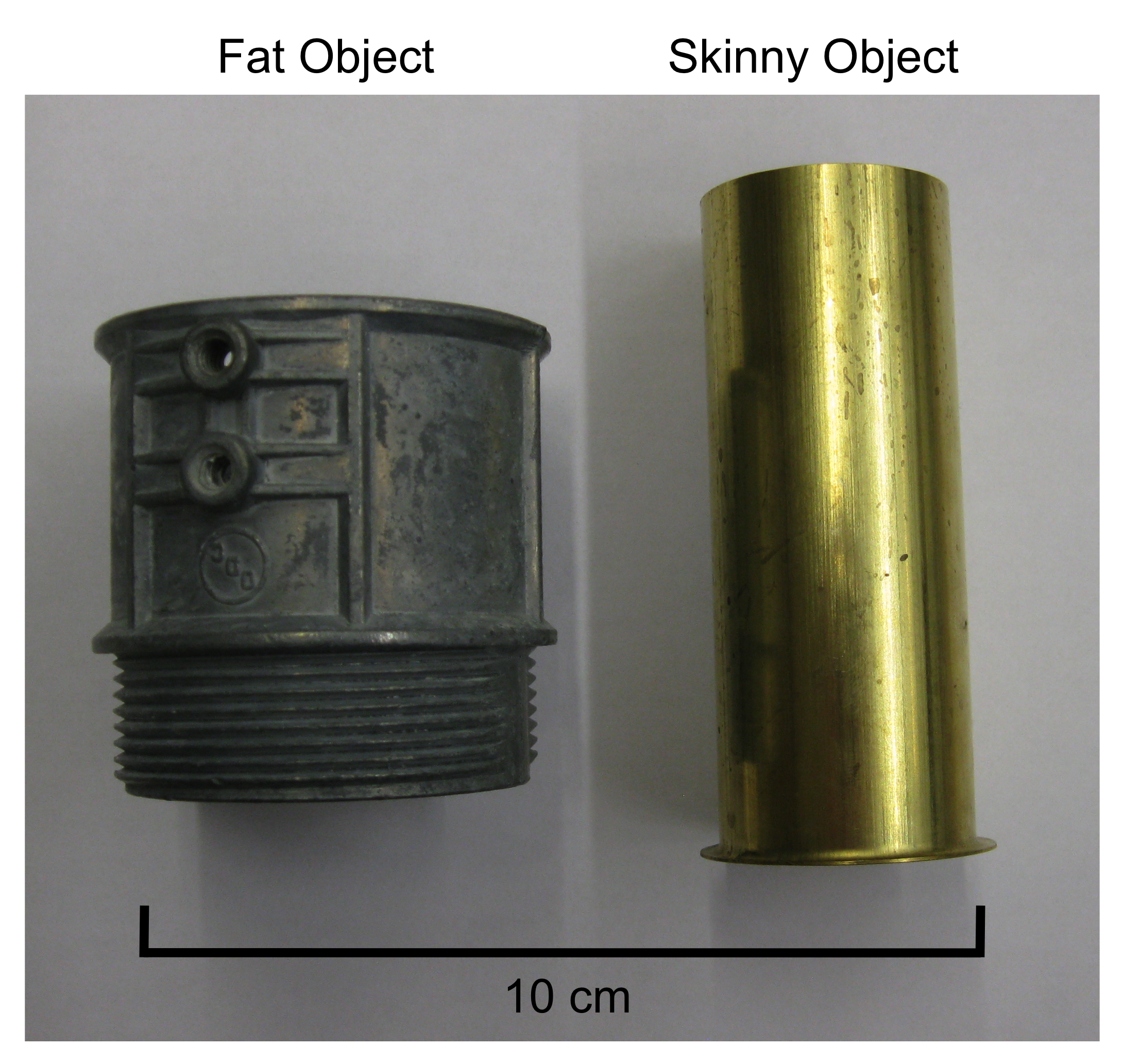

Supplement: Figure S1 — Digital photograph of the two novel objects presented to experimental fish in the current study. The so-called ‘fat object’ was a 8 × 6 cm (height × width) steel cylinder, and the ‘skinny object’ was a 10 × 4 cm brass cylinder. (TIFF) [file pone.0075858.s002.tiff]

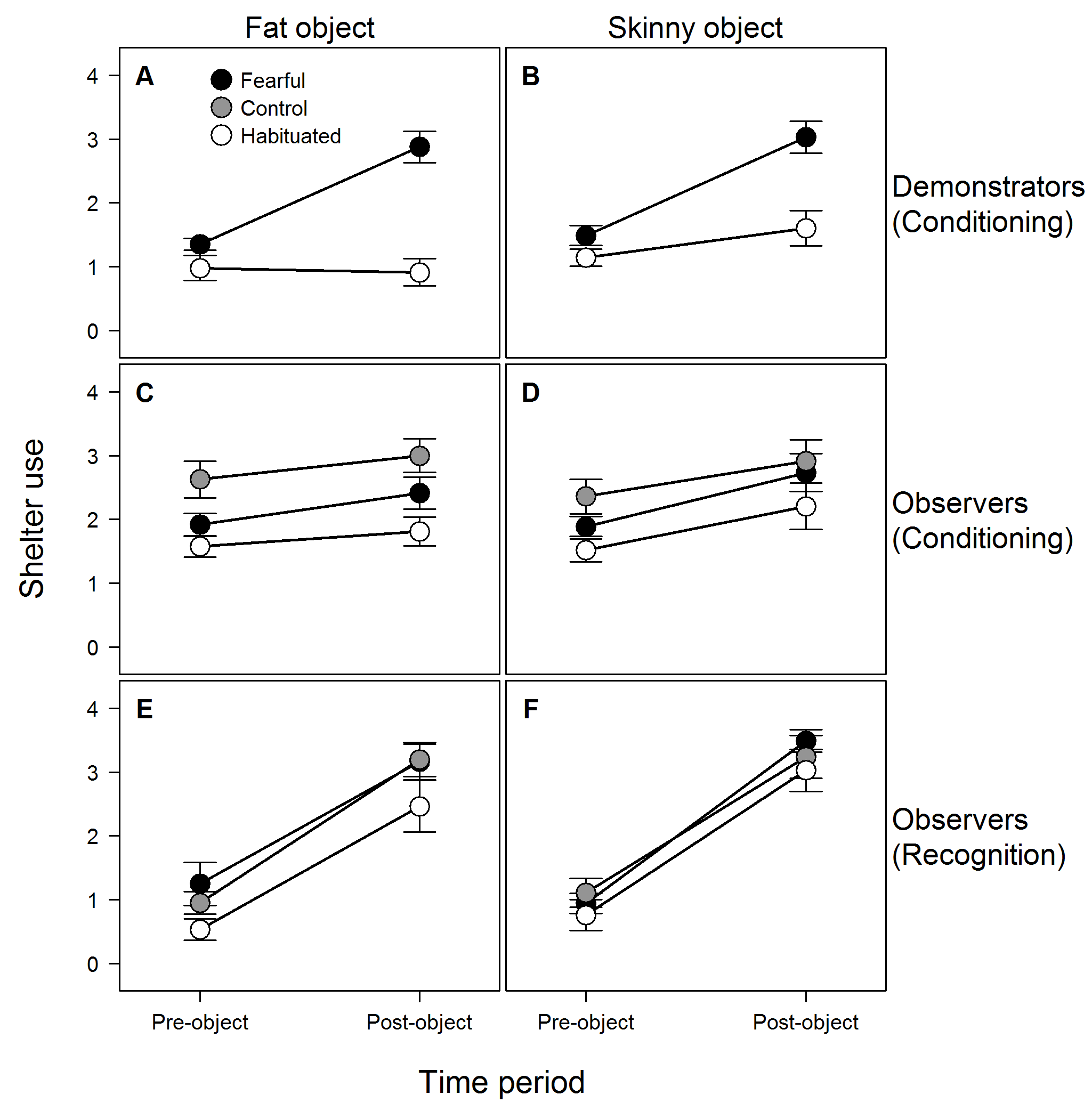

Supplement: Figure S3 — Changes in shelter use in response to a novel object, based on 1-minute pre- and post-object periods. Mean (± SE) shelter use by demonstrators during the conditioning phase (A, B) and the corresponding observers during either the conditioning phase (C, D) or recognition phase (E, F). During conditioning, demonstrators that had previously been either habituated to (open circles) or made fearful of (black circles) either the fat (A) or skinny (B) object were presented with the same object that they had been trained with while observers (C, D) witnessed their behaviour from adjacent aquaria. In the recognition phase, observers that, during conditioning, had witnessed either fearful demonstrators (black circles), no demonstrators (grey circles), or habituated demonstrators (open circles) reacting to either the fat (E) or skinny (F) object, were all presented with the skinny object. In each panel, pre-object scores reflect behaviour during the 1-min period prior to presentation of an object, whereas post-object scores reflect behaviour during the 1-min period following object presentation. (TIFF) [file pone.0075858.s004.tiff]

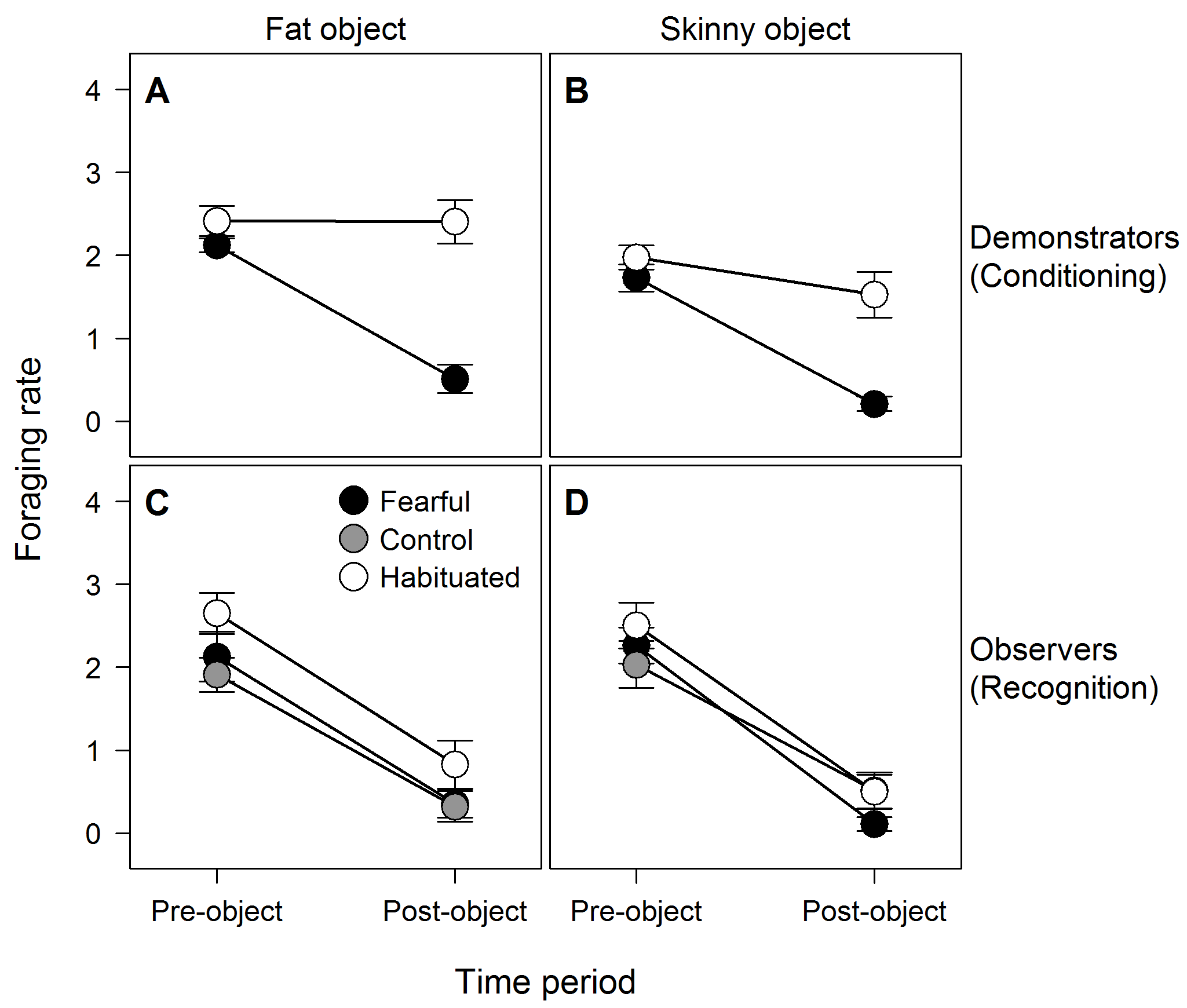

Supplement: Figure S4 — Changes in foraging rate in response to a novel object, based on 1-minute pre- and post-object periods. Mean (± SE) foraging rate of demonstrators during the conditioning phase (A, B) and the corresponding observers during the recognition phase (C, D). During conditioning, demonstrators that had previously been either habituated to (open circles) or made fearful of (black circles) either the fat (A) or skinny (B) object were presented with the same object that they had been trained with while observers witnessed their behaviour from adjacent aquaria. In the recognition phase, observers that, during conditioning, had witnessed either fearful demonstrators (black circles), no demonstrators (grey circles), or habituated demonstrators (open circles) reacting to either the fat (C) or skinny (D) object, were all presented with the skinny object. In each panel, pre-object scores reflect behaviour during the 1-min period prior to presentation of an object while post-object scores reflect behaviour during the 1-min period following object presentation. (TIFF) [file pone.0075858.s005.tiff]

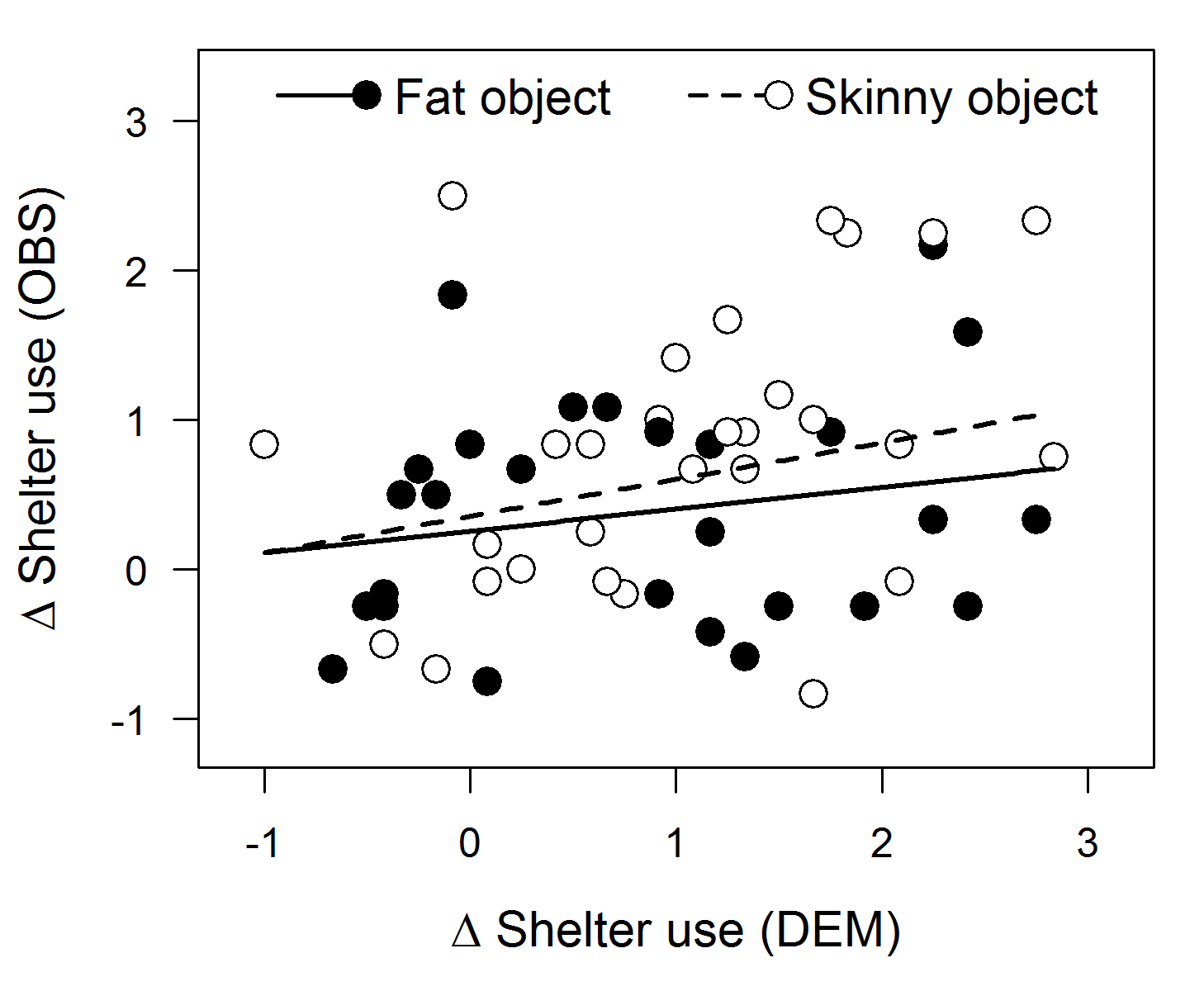

Supplement: Figure S5 — Relationship between the behaviour of demonstrator and observers during the conditioning phase, based on 1-minute pre- and post-object periods. Relationship between the change (Δ) in shelter use of observers (OBS) and their corresponding demonstrators (DEM) during the conditioning phase. The depicted relationships are based on all trials for which there were demonstrators (i.e. habituated and fearful treatments combined, n = 60). Scores for groups exposed to the fat object during the conditioning phase are shown as black circles (and solid regression lines), whereas scores for those exposed to the skinny object during the conditioning phase are depicted as open circles (and dashed regression lines). (TIFF) [file pone.0075858.s006.tiff]

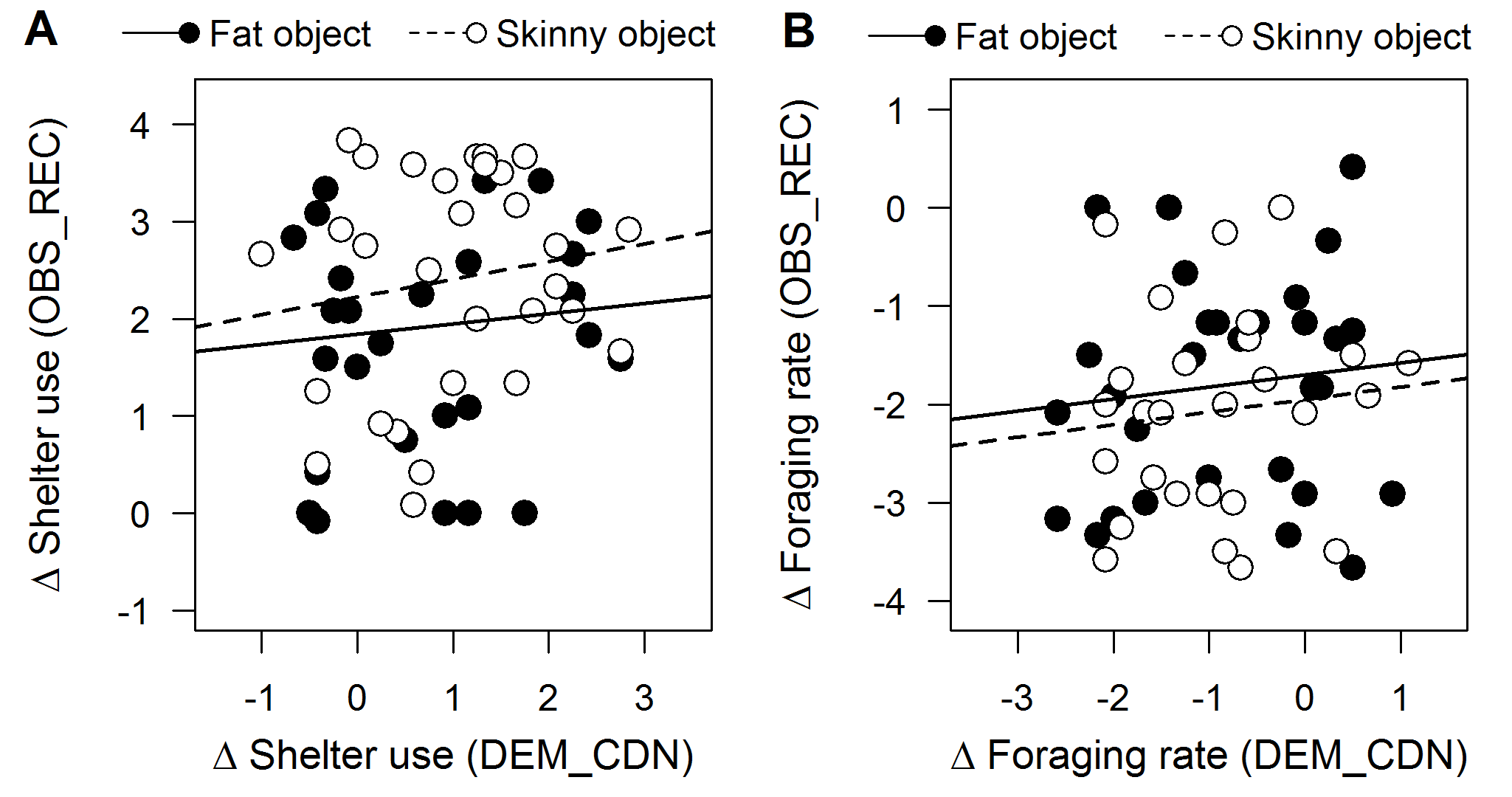

Supplement: Figure S6 — Relationship between the behaviour of demonstrators and observers during the conditioning and recognition phases, respectively, based on 1-minute pre- and post-object periods. Relationship between the change (Δ) in shelter use (A) and foraging rate (B) of observers during the recognition phase (OBS_REC) and that of the corresponding demonstrators during the conditioning phase (DEM_CDN). The depicted relationships are based on all trials for which there were demonstrators (i.e. habituated and fearful treatments combined, n = 60). Scores for groups exposed to the fat object during the conditioning phase are shown as black circles (and solid regression lines), whereas scores for those exposed to the skinny object during the conditioning phase are depicted as open circles (and dashed regression lines). (TIFF) [file pone.0075858.s007.tiff]
